# Species-Specificity of the BamA Component of the Bacterial Outer Membrane Protein-Assembly Machinery

**DOI:** 10.1371/journal.pone.0085799

**Published:** 2013-12-20

**Authors:** Elena B. Volokhina, Jan Grijpstra, Frank Beckers, Erika Lindh, Viviane Robert, Jan Tommassen, Martine P. Bos

**Affiliations:** Department of Molecular Microbiology and Institute of Biomembranes, Utrecht University, Utrecht, The Netherlands; Monash University, Australia

## Abstract

The BamA protein is the key component of the Bam complex, the assembly machinery for outer membrane proteins (OMP) in gram-negative bacteria. We previously demonstrated that BamA recognizes its OMP substrates in a species-specific manner *in vitro*. In this work, we further studied species specificity *in vivo* by testing the functioning of BamA homologs of the proteobacteria *Neisseria meningitidis*, *Neisseria gonorrhoeae*, *Bordetella pertussis*, *Burkholderia mallei*, and *Escherichia coli* in *E. coli* and in *N. meningitidis*. We found that no BamA functioned in another species than the authentic one, except for *N. gonorrhoeae* BamA, which fully complemented a *N. meningitidis bamA* mutant. *E. coli* BamA was not assembled into the *N. meningitidis* outer membrane. In contrast, the *N. meningitidis* BamA protein was assembled into the outer membrane of *E. coli* to a significant extent and also associated with BamD, an essential accessory lipoprotein of the Bam complex.Various chimeras comprising swapped N-terminal periplasmic and C-terminal membrane-embedded domains of *N. meningitidis* and *E. coli* BamA proteins were also not functional in either host, although some of them were inserted in the OM suggesting that the two domains of BamA need to be compatible in order to function. Furthermore, conformational analysis of chimeric proteins provided evidence for a 16-stranded β-barrel conformation of the membrane-embedded domain of BamA.

## Introduction

Gram-negative bacteria are characterized by a cell envelope consisting of an inner and an outer membrane (OM), which are separated by the peptidoglycan-containing periplasm. While integral inner membrane proteins are α-helical, all but two known integral OM proteins (OMPs) are β-barrels [1–3]. Only recently, the mechanisms of the assembly of OMPs into the OM have started to come to light. Previously, we showed that the Omp85 protein is an essential part of the OMP assembly machinery in *Neisseria meningitidis*; depletion of this essential protein in a conditional mutant strain resulted in the accumulation of unassembled forms of all OMPs analyzed [4]. Later, a similar function was shown for the Omp85 homologs in *Escherichia coli* [5-7], *Pseudomonas aeruginosa* [8] and *Borrelia burgdorferi* [9]. Omp85 homologs have been renamed BamA, for β-barrel assembly machine component A. Interestingly, a BamA homolog is also present and required for the assembly of β-barrel OMPs in mitochondria [10-12]. In *E. coli*, four lipoproteins associated with BamA (*Ec*BamA) have been identified: BamB, BamC, BamD, and BamE [6,13]. We established that *N. meningitidis* BamA (*Nm*BamA) is also associated with BamC, BamD (ComL) and BamE, and, additionally, with the RmpM protein [14]. BamB is not present in neisserial strains. 

BamA was predicted to consist of an N-terminal part localized in the periplasm and a membrane-embedded β-barrel in its C-terminal part [4]. In the predicted periplasmic part, five polypeptide-transport-associated (POTRA) domains are located [15]. Crystallographic and NMR structural studies have shown a similar fold for all POTRA domains: a β-sheet of three β-strands overlaid with a pair of antiparallel α-helices [16-19]. For the β-barrel domain, originally a 12-stranded β-barrel topology was predicted [4], which leaves a 61-amino-acid region between the POTRA and β-barrel domains, which we designated the hinge region [20]. However, the crystal structure of the two-partner secretion-system component FhaC of *Bordetella pertussis*, which shares limited sequence similarity with BamA, showed that this protein consists of two POTRA domains and a 16-stranded β-barrel, suggesting that the BamA β-barrel might also contain 16 β-strands, with four additional β-strands located in the postulated hinge region [21,22].

We have shown that *Ec*BamA interacts with OMPs via the so-called signature sequence present at the C terminus of the substrates [23]. This sequence is characterized by a phenylalanine or a tryptophan at the C-terminal position and hydrophobic amino-acid residues at positions -3, -5, -7, and -9 from the C terminus [24]. *Ec*BamA interacted *in vitro* with *E. coli*-derived C-terminal signature sequences, but not with those found in neisserial OMPs, the defining difference being the nature of the penultimate C-terminal amino acid [23]. These results are consistent with the observation that neisserial OMPs are not efficiently assembled in *E. coli*. This apparently high species-specificity of the Bam machinery seems to be at odds with the observation that the Bam machinery of *E. coli* can properly assemble a mitochondrial β-barrel OMP [25] and with the finding that bacterial OMPs were properly assembled into the mitochondrial OM of *Saccharomyces cerevisiae* [26]. To gain further insight into the species-specificity of the functioning of the Bam machinery, we investigated here whether BamA homologs from various Gram-negative bacteria are able to substitute each other *in vivo*. 

## Materials and Methods

### Bacterial Strains and Growth Conditions

*E. coli* strains TOP10F' (Invitrogen), DH5α (laboratory collection), and the *Ec*BamA-depletion strain UTP_BAD_::*bamA* [27] were grown either on Luria-Bertani (LB) agar plates or in liquid LB medium on a shaker at the indicated temperatures. When necessary, the media were supplemented with an appropriate antibiotic (25 μg/ml chloramphenicol or 50 μg/ml kanamycin), 0.4% glucose, 0.02% L-arabinose or 0.5 mM isopropyl-β-D-thiogalactopyranoside (IPTG). *N. meningitidis* strain HB-1[28], an unencapsulated derivative of the serogroup B strain H44/76, and *Neisseria gonorrhoeae* strain FA1090 from our laboratory collection were grown at 37°C in candle jars on GC agar plates (Oxoid), supplemented with Vitox (Oxoid), and, when necessary, with an antibiotic (10 μg/ml chloramphenicol or 80 μg/ml kanamycin). Liquid cultures were obtained by growing *N. meningitidis* for 6 h in tryptic soy broth (Becton Dickinson) in the absence or presence of 1 mM IPTG. 

### Plasmid Constructions

 The plasmids and primers used are listed in Tables S1 and S2, respectively. DNA fragments containing *bamA* genes were obtained by PCR using genomic DNA from *N. meningitidis* HB-1 plus primer pair NmF/NmR, *N. gonorrhoeae* FA1090 DNA plus primer pair NmF/NmR , *Burkholderia mallei* Bogor DNA (provided by the Central Veterinary Institute, Lelystad, The Netherlands) plus primer pair BmF/BmR, *Bordetella pertussis* Tohama I DNA (provided by the Netherlands Vaccine Institute, Bilthoven, The Netherlands) plus primer pair BpF/BpR and *E. coli* DH5α DNA plus primer pair EcF/EcR, and cloned into pCRII-TOPO. We used the sequences of the locus tags NMB0182, NGO1801, BMA1547, BP1427 and b0177, respectively (www.ncbi.nlm.nih.gov/gene), to design primers. The primers carried restriction sites allowing for subcloning of the genes behind the IPTG-inducible promoter on plasmid pFP10-c-*lbpA*, which is able to replicate in *N. meningitidis* as well as in *E. coli*. As a result of the cloning strategy, the original signal sequences were substituted by that of the lactoferrin-binding protein A (LbpA) of *N. meningitidis* (MNKKHSFPLTLTALAIATAFPSYA). The *Nde*I-*Aat*II fragments, carrying *N. meningitidis*, *N. gonorrhoeae*, and *E. coli bamA* genes were ligated into *Nde*I-*Aat*II-digested pFP10-c-*lbpA*, yielding pFP10-Nm*bamA*, pFP10-Ng*bamA*, and pFP10-Ec*bamA*, respectively. The fragments containing *B. pertussis* and *B. mallei bamA* were excised using the *Nde*I site introduced by the forward primers and the *Kpn*I site of pCRII-TOPO and introduced into *Nde*I-*Kpn*I-digested pFP10-Ec*bamA*, yielding pFP10-Bp*bamA* and pFP10-Bm*bamA*, respectively. 

A construct encoding the C-terminal 317 amino acids of *Nm*BamA fused to the signal sequence of LbpA was created by PCR using primer pair Omp85-F481-NmR and pFP10-Nm*bamA* as template. The resulting PCR product was cloned into pCRII-TOPO and ligated into *Nde*I-*Aat*II-digested pFP10-c-*lbpA*, yielding pFP10-_481_Nm*bamA.*

The N- and C-terminal domains of *Nm*BamA and *Ec*BamA were swapped resulting in chimeras. Two chimeric *bamA* genes were designed encoding proteins in which the N-terminal and C-terminal domains of *Nm*BamAand *Ec*BamA were exchanged within a small region of high homology around the start of the predicted 12-stranded β-barrel [4]. The construct designated *Ec*_*479*_ *Nm* encodes a protein carrying the N-terminal part of *Ec*BamA and the C-terminal part of *Nm*BamA, while construct *Nm*_*480*_ *Ec* encodes the reverse chimera. The numbers indicate the most C-terminal amino acid of the BamA domain present in the N-terminal part of the chimera (counting the N-terminal signal sequence). For the *Ec*_*479*_ *Nm* construct, two overlapping PCR products were made: one using the EcF and HingeR primers and pFP10-Ec*bamA* as template and the other using the HingeF and NmR primers and pFP10-Nm*bamA* as template. The purified PCR products were mixed, melted and annealed and combined with EcF and NmR as external primers in a second PCR to obtain the complete chimeric genes. For the *Nm*_*480*_ *Ec* chimera, also two PCR products were generated: one using NmF and HingeR as primers and pFP10-Nm*bamA* as template, and the other with HingeF and EcR as primers and pFP10-Ec*bamA* as template. The second PCR was performed using purified PCR products and NmF and EcR as external primers. The obtained chimeras were introduced into pCRII-TOPO. The *Nde*I-*Aat*II fragments were subsequently ligated into pFP10-c-*lbpA* digested with *Nde*I and *Aat*II, yielding constructs pFP10-*Ec*_*479*_ *Nm* and pFP10-*Nm*_*480*_ *Ec*.

Another set of chimeras, designated *Ec*_*423*_ *Nm* and *Nm*_*423*_ *Ec*, was constructed to produce hybrid proteins where domain exchange occurred at the C terminus of the predicted POTRA5 domain [18] with the N-terminal domain of the chimeras comprising 423 amino-acid residues. To make the *Ec*_*423*_ *Nm* construct, a megaprimer was created using primers EcNmF and Omp85R2, and pFP10-Nm*bamA* as the template. The purified megaprimer was combined with the EcF primer and pFP10-Ec*bamA* as the template for a second PCR and the product was cloned into pCRII-TOPO. The chimeric fragment was then introduced into pFP10-Nm*bamA*, using the *Nde*I site and the *Kpn*I site present in the *N. meningitidis bamA* gene, yielding pFP10-*Ec*_*423*_ *Nm*. For the *Nm*_*423*_ *Ec* chimera, megaprimer1 was generated using primer pair NmEcR-Omp85F3 and pFP10-Nm*bamA* as the template. For the second PCR, megaprimer1 was combined with the EcR primer and pFP10-Ec*bamA* as the template. The chimeric fragment obtained was cloned into pCRII-TOPO, yielding pCRII-*Nm*_*423*_ *Ec-a*. Megaprimer2 was produced using primers Omp85F4 and Omp85R7 and pFP10-Nm*bamA* as template. Megaprimer2 was then combined with EcR and pCRII-*Nm*_*423*_ *Ec-a* as template in a PCR. The resulting PCR product was cloned into pCRII-TOPO, yielding pCRII-*Nm*_*423*_ *Ec-b*. The missing 5' part of Nm*bamA*was added through insertion of a *Sal*I-*Not*I fragment of pCRII-Nm*bamA*, a precursor of pFP10-Nm*bamA*, into *Sal*I-*Not*I-restricted pCRII-*Nm*_*423*_ *Ec-b*, yielding pCRII-*Nm*_*423*_ *Ec-c*. Finally, an *Nde*I-*Kpn*I fragment of pCRII-*Nm*_*423*_ *Ec-c* was ligated into *Nde*I-*Kpn*I-digested pFP10-Ec*bamA*, yielding pFP10-*Nm*_*423*_ *Ec*. Plasmid pRV-His-*Nm*BamA was created by insertion of a *Not*I-*Aat*II fragment, encoding *Nm*BamA with an additional HHHHHHQDF amino-acid sequence between the signal sequence and the N terminus of the mature BamA protein, from pEN11-His-Omp85 into *Not*I-*Aat*II restricted pRV2000. All constructs were verified by sequencing.

### RT-PCR

 Reverse transcription-PCR (RT-PCR) was performed as described [29] except that conventional PCR instead of real-time PCR was performed for amplification of cDNA. Primer couples Q-for-1/Q-rev-Ngo, Q-for-2/Q-rev-Eco, Q-for-3/Q-rev-Bper and Q-for-1/Q-rev-Bmal were used to amplify the *bamA* cDNAs of *N. gonorrhoeae, E. coli, B. pertussis* and *B. mallei*, respectively.

### Complementation Assays

 To test whether various BamA variants could complement BamA deficiency in *E. coli*, pFP10-based plasmids containing *bamA* variants under the control of an IPTG-inducible promoter were introduced into *Ec*BamA-depletion strain UTP_BAD_::*bamA*, which produces *Ec*BamA from an arabinose-inducible promoter. Growth of the resultant strains in LB with indicated supplements was assessed at 37°C or on LB plates at 22°C. To evaluate BamA complementation in *N. meningitidis*, it was tested whether the chromosomal copy of *bamA* could be disrupted, when *bamA* variants were expressed from the pFP10-derived plasmids. To this end, pRV1300 was used as the template to generate a PCR product containing a fragment upstream of *N. meningitidis bamA*, a kanamycin-resistance cassette and a 3' fragment of *bamA*, as described [4]. The PCR fragment was used to transform HB-1 cells carrying *bamA* variants on plasmid. Transformations were done in the presence of IPTG and transformants were selected on GC agar plates supplemented with 1 mM IPTG and kanamycin and analyzed by PCR using primer pair G and Omp85R2 that hybridize upstream and in the 3' part of Nm*bamA*. 

### Isolation of Cell Envelopes

 To isolate cell envelopes, bacteria from liquid cultures were collected by centrifugation, resuspended in 50 mM Tris-HCl, 5 mM EDTA (pH 8.0) containing protease inhibitor cocktail “Complete” (Roche) and stored overnight at -80°C. After ultrasonic disintegration (3 x 45 s at level 8, output 40%, Branson sonifier 450; Branson Ultrasonics Corporation), unbroken cells were removed by centrifugation (12,000 *g*, 15 min, 4°C). Cell envelopes were collected by ultracentrifugation (170,000 x *g*, 5 min, 4°C), dissolved in 2 mM Tris-HCl (pH 7.6) and stored at –20°C.

### Urea Extraction

 Thirty-μl samples of cell envelope preparations were incubated in 1 ml of 20 mM Tris-HCl, 100 mM glycine (pH 7.6), 6 M urea for 1 h at room temperature while rotating. The insoluble material was separated from the soluble fraction by ultracentrifugation (200,000 x *g*, 1 h, 4°C). The pellet was dissolved in 30 μl of 2 mM Tris-HCl (pH 7.6), while the solubilized proteins were precipitated from the supernatant with 10% trichloroacetic acid (TCA) and dissolved in 30 μl H_2_O.

### Trypsin Treatment

 Exponentially growing cells were pelleted and resuspended in phosphate-buffered saline plus 1 mM MgCl_2_ and 0.5 mM CaCl_2_ to an optical density at 550 nm (OD_550_) of 2. Trypsin was added to 200-µl portions of this suspension. After incubation for 15 min at room temperature, 1 mM phenylmethanesulfonyl fluoride (PMSF) was added, and the bacteria were collected by centrifugation and boiled in sample buffer for sodium dodecyl sulfate-polyacrylamide gel electrophoresis (SDS-PAGE). Cell envelopes were treated with 50 μg/ml trypsin at room temperature for the indicated time periods and subsequently boiled in SDS-PAGE sample buffer.

### SDS-PAGE and Immunoblotting

 Proteins were analyzed by standard denaturing SDS-PAGE as described [14], followed by staining with Coomassie Brilliant Blue or silver [30], or immunoblotting as described [14]. *Ec*BamA was detected with a rabbit antiserum raised against the denatured full-length protein [23]. Rabbit antisera against the N-terminal (residues 22-464) and C-terminal (residues 455-797) regions of *N. meningitidis* BamA (α-N-*Nm*BAmA and α-C-*Nm*BamA, respectively) were generously provided by Ralph Judd (University of Montana, USA). The antisera against *E. coli* BamD and BamB were generous gifts of Naoko Yokota and Hajime Tokuda (University of Tokyo, Japan). A monoclonal antibody (Mab) directed against the POTRA1 domain of *Nm*BamA (α-POTRA1_Nm_) came from GlaxoSmithKline Biologicals (Rixensart, Belgium).

### Affinity Purification

 Extraction and purification of His-tagged BamA from cell envelopes using Ni^2+^-nitrilotriacetic acid (Ni^2+^-NTA)-agarose beads (Qiagen) was performed as described [14].

## Results

### *E. coli* BamA does not function in *N. meningitidis*

**Figure 1:**
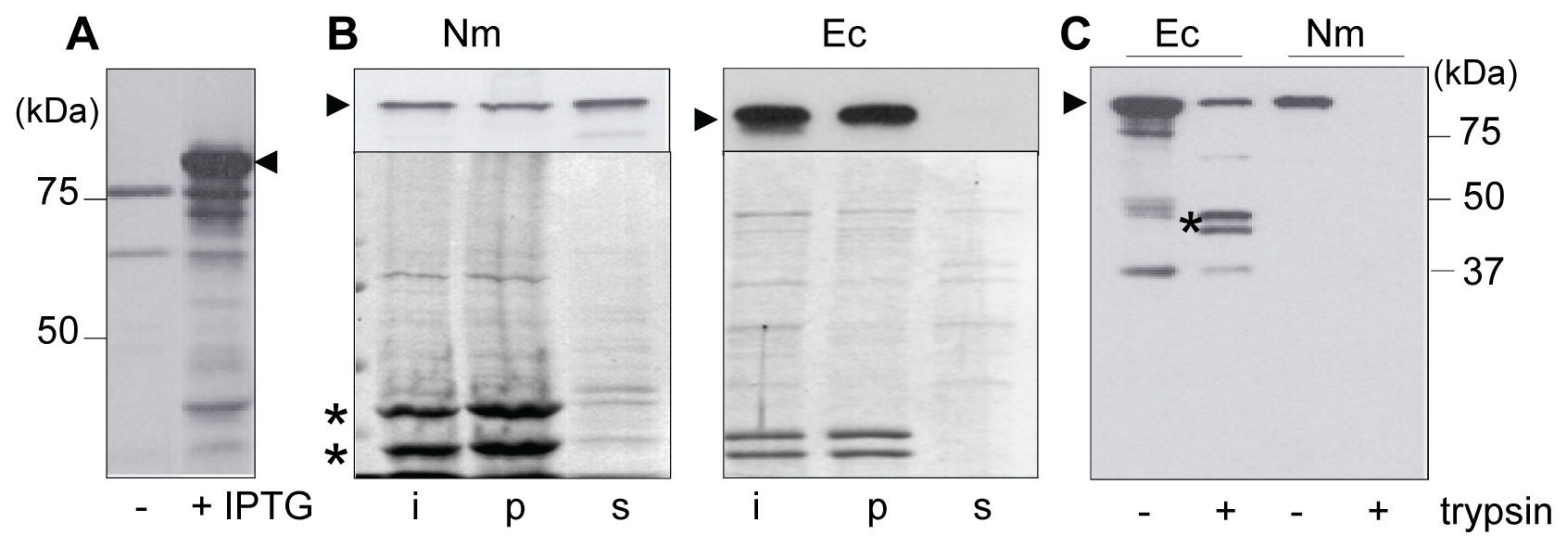
Expression and assembly of *E. coli* BamA in *N. meningitidis*. Cell envelopes were analyzed by SDS-PAGE followed by immunoblotting using anti-*Ec*BamA antiserum or by staining with Coomassie Brilliant Blue. BamA is indicated with arrowheads. A: Immunoblots of cell envelopes of uninduced (-) or induced (+) HB-1 cells containing pFP10-Ec*bamA* carrying *bamA* under an IPTG-inducible promoter. B: Cell envelopes of HB-1 (Nm) expressing *E*cBamA or of *E. coli* strain DH5α (Ec) were extracted with urea and the input (i), urea-insoluble (p) and -soluble (s) fractions were analyzed by SDS-PAGE followed by staining with Coomassie Brilliant Blue (lower panels) or immunoblotting with anti-*Ec*BamA antiserum (upper panels). Neisserial porins are indicated with asterisks. C: Cell envelopes of *E. coli* strain DH5α (Ec) or HB-1 (Nm) expressing *Ec*BamA were treated (+) or not (-) with trypsin for 1 h and analyzed by SDS-PAGE followed by immunoblotting with anti-*Ec*BamA antiserum. Trypsin-protected *Ec*BamA fragments in *E. coli* are indicated with an asterisk.

 To determine whether *E. coli* BamA could substitute for *N. meningitidis* BamA *in vivo*, a plasmid containing the Ec*bamA* gene under control of a *lac* promoter was introduced into *N. meningitidis* strain HB-1. This resulted in IPTG-dependent expression of Ec*bamA* in HB-1 (Figure 1A). As a control, HB-1 was also transformed with a similar plasmid containing *N. meningitidis bamA*. Next, we attempted to disrupt the chromosomal Nm*bamA* copy in the transformants. Correct mutants were easily obtained when *N. meningitidis bamA* was expressed from plasmid. In contrast, PCR analysis showed that the few kanamycin-resistant transformants that were obtained from the cells expressing Ec*bamA* still contained the wild-type copy of Nm*bamA* on the chromosome. Apparently, *Ec*BamA cannot replace *N. meningitidis* BamA. 

The inability of *Ec*BamA to substitute for *Nm*BamA could be due to its failure to become correctly assembled in the meningococcal OM. To test this hypothesis, we analyzed the extractability of *Ec*BamA with urea from these membranes. Correctly inserted OMPs are not usually extracted with urea, as demonstrated for the neisserial porins in Figure 1B (left panel). However, most of *Ec*BamA present in neisserial cell envelopes was extractable with urea (Figure 1B, left panel), whereas, as expected, this was not the case for *Ec*BamA in *E. coli* cell envelopes (Figure 1B, right panel). Correctly assembled β-barrel domains of OMPs are also usually resistant to proteases. Accordingly, the intact C-terminal β-barrel domain of *Ec*BamA can be obtained by treating *E. coli* cell envelopes with trypsin [23] (Figure 1C). However, when neisserial cell envelopes containing *Ec*BamA were treated with trypsin, no protected fragment was found (Figure 1C), indicating that no part of *Ec*BamA is properly assembled into the OM. Together, these data demonstrate that *Ec*BamA, for the most part, was not inserted in the neisserial OM and, therefore, cannot be expected to functionally replace *Nm*BamA.

### *N. meningitidis* BamA does not function in *E.coli*

**Figure 2:**
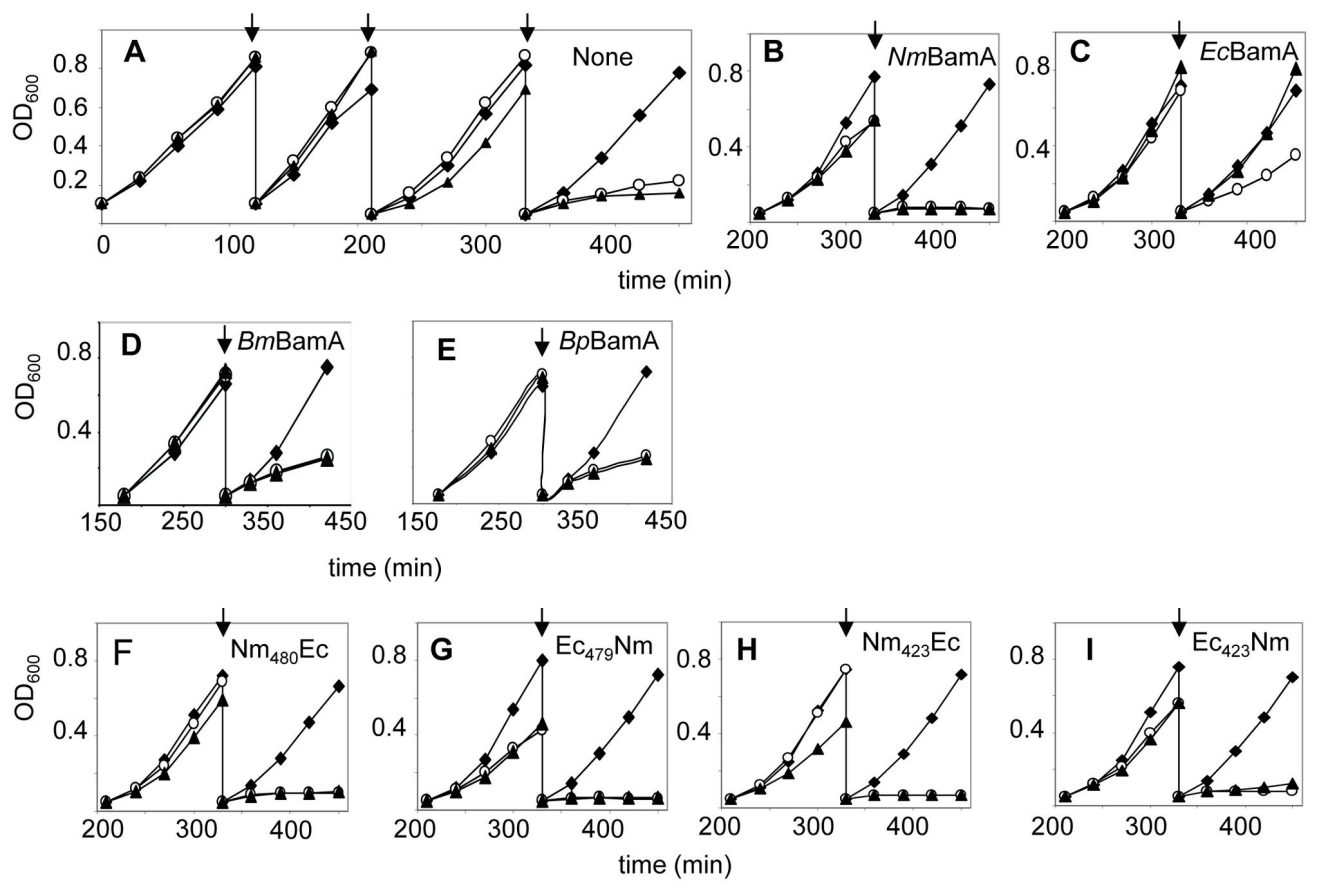
Functionality of BamA variants in *E. coli*. Growth of *E. coli* strain UTP_BAD_::*bamA* carrying no plasmid (panel A) or plasmids encoding the proteins indicated in the upper right hand side of the graphs was assessed by measuring the OD_600_. Strains were grown in the presence of glucose (open circles), arabinose (closed diamonds) or IPTG (closed triangles) in LB at 37°C. Cultures were diluted into fresh medium at the time points indicated by arrows.

**Figure 3:**
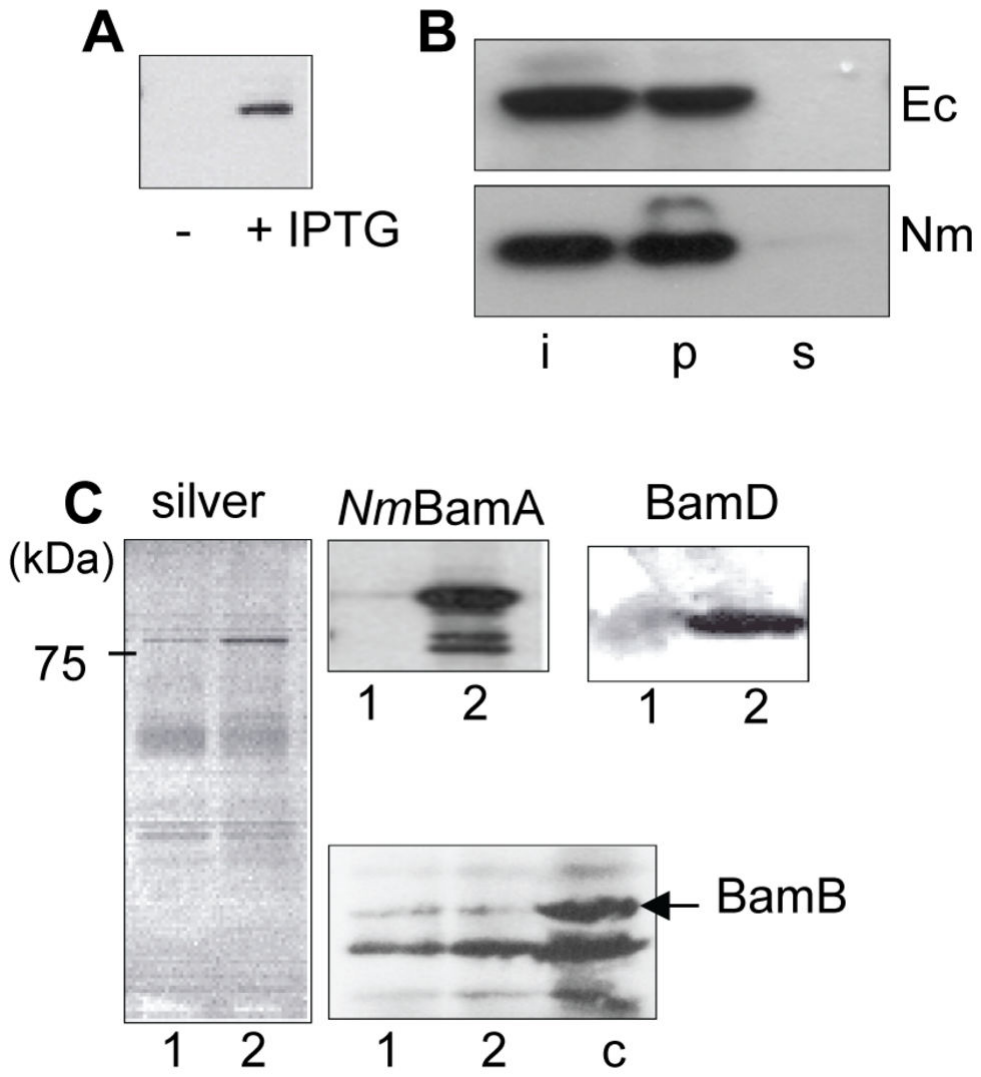
Expression and assembly of *N. meningitidis* BamA in *E. coli*. A: Expression of Nm*bamA* in *E. coli*. *E. coli* strain UTP_BAD_::*bamA* containing pFP10-Nm*bamA* was grown in LB containing 0.02% arabinose in the absence or presence of IPTG. Cell envelopes were isolated and analyzed by immunoblotting with Mab α-POTRA1_Nm_. B: Assembly of *Nm*BamA in the *E. coli* OM. Cell envelopes from *E. coli* strain UTP_BAD_::*bamA* containing pFP10-Nm*bamA*grown in LB containing 0.02% arabinose and IPTG were extracted with urea (Ec). As a control, cell envelopes of strain HB-1 were extracted with urea (Nm). Input (i), urea-insoluble (p) and -soluble (s) fractions were analyzed by SDS-PAGE and immunoblot analysis with Mab α-POTRA1_Nm_. C: Co-purification of Bam-complex components with His-tagged *Nm*BamA in *E. coli*. Cell envelopes of *E. coli* DH5α cells containing pRV-His-Nm*bamA* either induced (lanes 2) or not (lanes 1) with IPTG were extracted with Elugent and subjected to Ni^2+^-NTA purification. Shown are elution fractions analyzed by denaturing SDS-PAGE and silver staining (left panel) or immunoblotting (right panels) using antisera against the indicated proteins. As a positive control for BamB detection, cell envelopes derived from strain DH5α were also analyzed on blot (lane c). The arrow indicates the position of BamB.

To assess whether *N. meningitidis* BamA can substitute for *E. coli* BamA, we made use of the *Ec*BamA-depletion strain UTP_BAD_::*bamA* in which chromosomal Ec*bamA* expression is under control of an arabinose-inducible promoter resulting in a growth-arrest after prolonged growth in the absence of arabinose (Figure 2A). Introduction of pFP10-Nm*bamA* into this strain did not rescue growth in the absence of arabinose even when *Nm*BamA synthesis was induced with IPTG (Figure 2B), while a control strain containing Ec*bamA* under IPTG control on pFP10-Ec*bamA* continued to grow under these conditions (Figure 2C). The expression of *N. meningitidis bamA* in response to IPTG induction was confirmed on immunoblots (Figure 3A). Even under conditions of slow growth, i.e. on LB plates at 22°C, *Nm*BamA did not support growth of *E. coli* (Figure S1). Thus, BamA of *N. meningitidis* does not function in *E. coli*. Interestingly, *N. meningitidis* BamA appeared to be inserted into the *E. coli* OM since it was not extracted from the membrane fraction with urea just as in neisserial cell envelopes (Figure 3B).

*Ec*BamA is associated with several lipoproteins, i.e., BamB, BamC, BamD and BamE [6,13]. A failure to associate with these lipoproteins might explain the lack of function of neisserial BamA in *E. coli*. To test this possibility, pRV-His-*Nm*BamA encoding *Nm*BamA with a His-tag at the N terminus of the mature protein was introduced into *E. coli* DH5α and expression of the recombinant protein was induced with IPTG. Cell envelopes were extracted with Elugent and the His-tagged *Nm*BamAwas affinity purified with Ni^2+^-NTA beads under native conditions (Figure 3C). A control purification was done on cells not producing the His-tagged *Nm*BamA. Remarkably, substantial amounts of *E. coli* BamD co-purified with the neisserial BamA (Figure 3C, lane 2), while no BamD was detected in the eluate of the control experiment with cells not expressing the His-tagged *Nm*BamA (Figure 3C, lane 1). In contrast to this essential lipoprotein, the non-essential lipoprotein BamB did not specifically co-purify with *Nm*BamA (Figure 3C). These results enforce the notion that at least a portion of *Nm*BamA produced in *E. coli* is correctly assembled.

### Complementation by Other BamA Homologs

**Figure 4:**
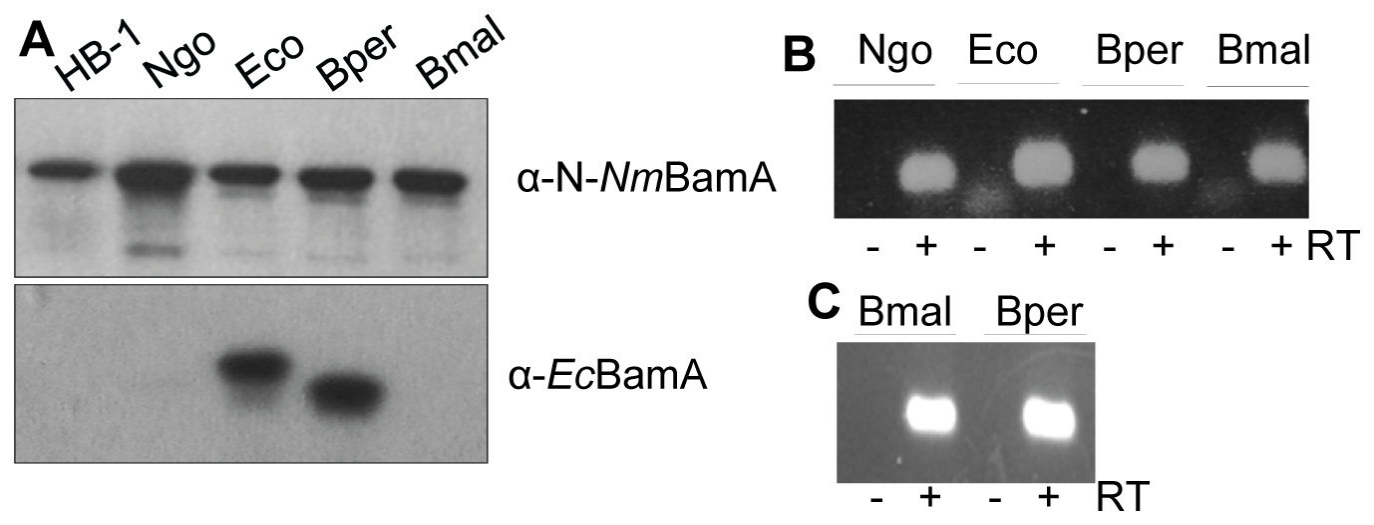
Expression of heterologous *bamA* variants in *N. meningitidis* and *E. coli*. A, B: *N. meningitidis* strain HB-1 and its derivatives were grown with IPTG to induce expression of *B. pertussis* (Bper), *B. mallei* (Bmal), *N. gonorrhoeae* (Ngo) and as control, *E. coli* (Eco) *bamA* genes from plasmids. A: Cell lysates were analyzed by SDS-PAGE and immunoblotting with the antisera indicated on the right. Note that also chromosome-encoded *Nm*BamA is detected with the α-N-*Nm*BamA antiserum in the upper panel. B: RNA was isolated and treated or not with reverse transcriptase (RT). Specific cDNA was detected by conventional PCR followed by agarose gel electrophoresis. The -RT samples serve as controls for the absence of plasmid DNA in the RNA preparations. C: RNA was isolated from UTP_BAD_::*bamA* containing pFP10-Bm*bamA* (Bmal) or pFP10-Bp*bamA* (Bper) grown in the presence of arabinose and IPTG and processed as explained for panel B.

 The observation that *Ec*BamA and *Nm*BamA could not functionally replace each other *in vivo* could be related to the evolutionary distance between *E. coli*, belonging to the class of γ-proteobacteria, and *N. meningitidis*, a β-proteobacterium. Therefore, we next investigated whether BamA from other β-proteobacteria, i.e. those of *B. pertussis, B. mallei* and *N. gonorrhoeae*, could functionally substitute BamA in *N. meningitidis*. Strain HB-1 was transformed with plasmids containing these *bamA* genes under *lac* promoter control. *N. gonorrhoeae* and *B. pertussis* BamA protein production could be verified by immunoblotting utilizing the apparent cross-reactivity of the α-N-*Nm*BamA and α-*Ec*BamA antisera, respectively (Figure 4A). Expression of the *B. mallei bamA* gene was demonstrated by RT-PCR (Figure 4B). Next, we tested whether the chromosomal *bamA* gene could be inactivated while the cells were kept in the presence of IPTG. We only succeeded to obtain correct mutants when the gonococcal *bamA* was expressed from plasmid. Thus, apparently, the meningococcal BamA protein can be functionally replaced by BamA of the closely related species *N. gonorrhoeae*, which shows 95% amino-acid sequence identity with the meningococcal one (Figure S2A), but not by those of *B. pertussis* or *B. mallei*, which are less similar (Figure S2B). We evaluated also whether the *B. pertussis* and *B. mallei* BamA proteins could substitute for *E. coli* BamA. Neither one allowed for growth of the *E.coli* BamA-depletion strain in the absence of arabinose and the presence of IPTG at 37°C (Figure 2D,E) or at 22°C (Figure S1) although they were expressed (Figure 4C), demonstrating that they cannot functionally substitute *Ec*BamA.

### Functionality of Chimeras of *N. meningitidis* and *E. coli* BamA

**Figure 5:**
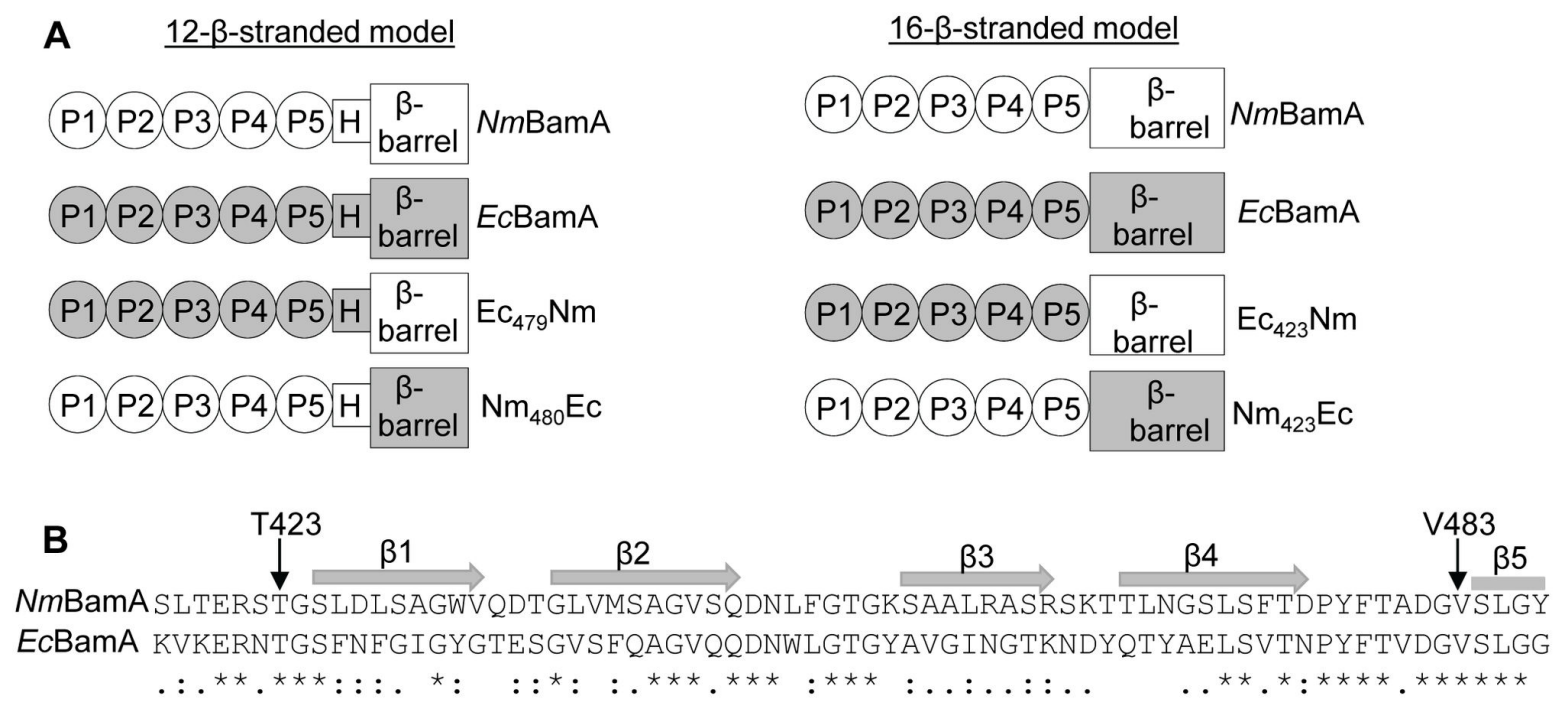
Schematic representation of the BamA chimeras used in this study. A: Two sets of chimeras were constructed based on two different topology models for BamA. In one model, the POTRA domains (P1-P5) are connected via a hinge region (H) to a 12-stranded β-barrel (left). In the other model, the POTRA domains are directly connected to a larger, 16-stranded β-barrel (right). *E. coli*-derived polypeptides are indicated in grey and *N. meningitidis* derived polypeptides in white. B: ClustalW alignment of partial sequences (residues 417-485, counting the signal sequence) of *N. meningitidis* and *E. coli* BamA. The C terminus of POTRA5 (T423) [18] and the N terminus of the predicted 12-stranded β-barrel (V483) [4] are indicated by arrows. The β-strands 1 through 4 plus the start of the fifth, forming the BamA β-barrel as determined in the recently published crystal structure of *Ng*BamA [37], are indicated with grey arrows.

**Figure 6:**
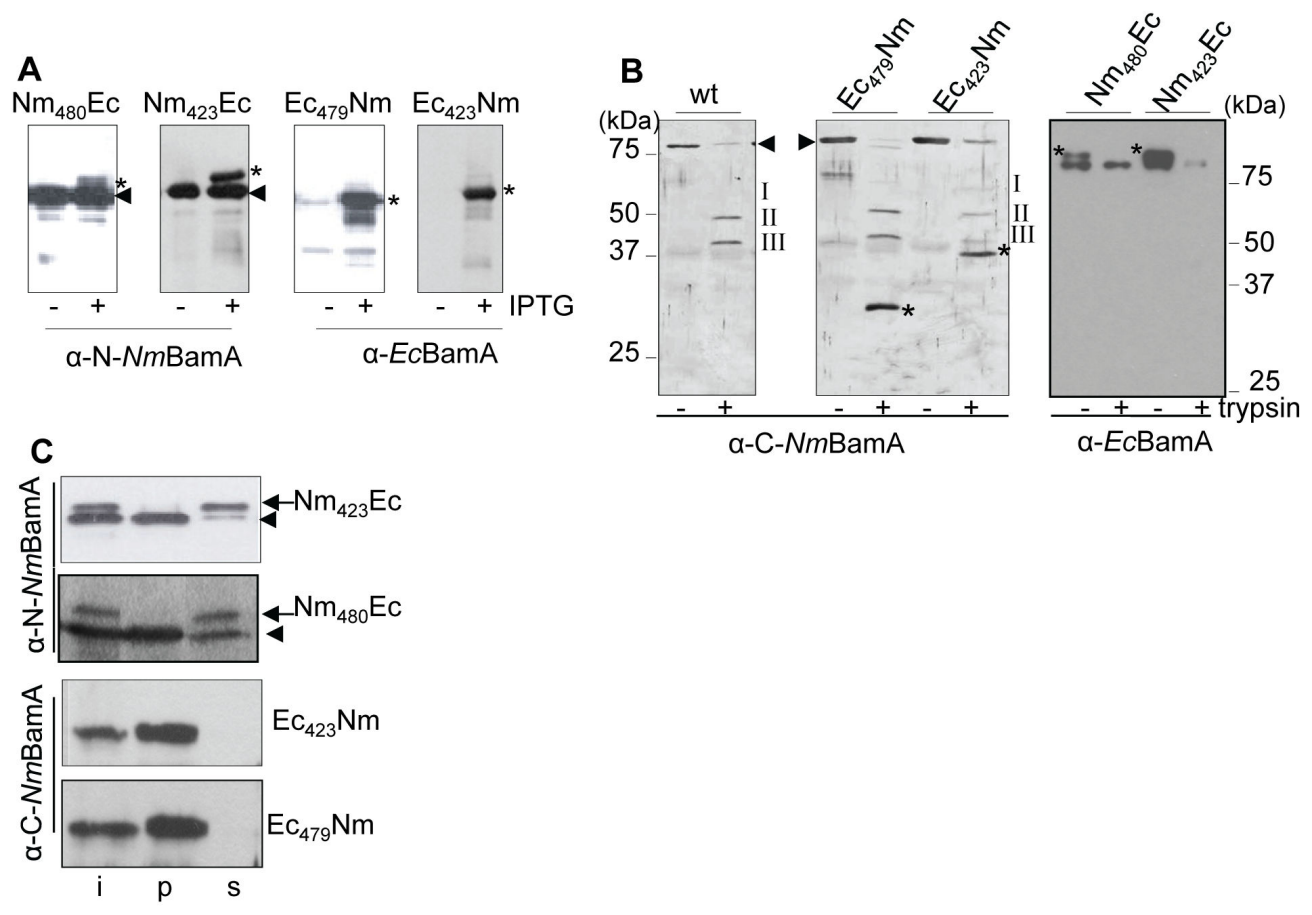
Expression and assembly of BamA chimeras in *N. meningitidis*. A: Derivatives of strain HB-1 containing plasmids encoding the chimeric proteins indicated above the panels were grown with or without IPTG. Cell envelopes were isolated and analyzed by SDS-PAGE and immunoblotting with the antisera shown below the blots. The chimeric proteins are indicated with asterisks. Note that also chromosome-encoded *Nm*BamA is detected with the α-N-*Nm*BamA antiserum (indicated with the arrowhead). B: Trypsin sensitivity of BamA variants in *N. meningitidis* cell envelopes. Cell envelopes of strain HB-1 expressing the indicated BamA variant were treated overnight with or without trypsin and analyzed on immunoblots. Chromosomally encoded *Nm*BamA in the parent strain HB-1 (wt) yielded three tryptic fragments indicated as I, II and III. Additional digestion products of Ec_479_Nm and Ec_423_Nm are indicated with asterisks. Arrowheads indicate the positions of undigested *Nm*BamA and the Ec_423_Nm and Ec_479_Nm chimeras, which all have indistinguishable electrophoretic mobilities. In the righ-hand panel, asterisks indicate the positions of undigested Nm_423_Ec and Nm_480_Ec. The blot required a long exposure time to visualize Nm_480_Ec which additionaly revealed a cross-reactive band just below the signal of the chimeras. C: Extractability of BamA variants from cell envelopes with urea. Cell envelopes of *N. meningitidis* strain HB-1 producing the chimeras indicated at the right were extracted with urea and the input (i), urea-insoluble (p) and -soluble (s) fractions were analyzed by SDS-PAGE and immunoblotting with the antibodies indicated on the left. Arrowheads indicate chromosomally encoded endogenous *Nm*BamA. In the lower two panels, the positions of endogenous BamA and the chimeric proteins are indistinguishable.

 To determine which part of the BamA protein could be dictating the species specificity of functionality, two sets of chimeras were constructed in which the N- and C-terminal domains of *Nm*BamA and *Ec*BamA are swapped. For the first couple of constructs, designated Ec_479_Nm and Nm_480_Ec, the site of the domain exchange was chosen based on a model predicting a 12-stranded β-barrel in the C-terminal part of BamA [4] (Figure 5A,B). The second set of chimeras, designated Ec_423_Nm and Nm_423_Ec, was designed based on a FhaC-like 16-stranded β-barrel [21]; in this case the domains were swapped directly after the C terminus of POTRA5 (Figure 5A,B). The chimeric genes were cloned behind an IPTG-inducible promoter and introduced into *N. meningitidis*. Synthesis of all hybrid proteins was detected when immunoblots with cell envelopes were probed with appropriate antisera (Figure 6A). However, the chromosomal copy of Nm*bamA* could not be inactivated during expression of any of the chimeras indicating that all four chimeras are non-functional in *N. meningitidis.*

To test whether the chimeric proteins were assembled in the OM, cell envelopes were treated overnight with trypsin. Such treatment of wild-type membranes yields three bands reactive with an antiserum directed against the C-terminal domain of *Nm*BamA indicated with I, II and III in Figure 6B [14]. Treatment of the membranes containing the Ec_423_Nm or Ec_479_Nm chimeras with trypsin also yielded bands I, II and III; this was expected since these membranes also contain native *Nm*BamA encoded by the chromosome. Remarkably, additional distinct fragments of 37 and 30 kDa were detected with the antiserum (indicated with asterisks in Figure 6B) indicating that the C-terminal β-barrel domain of these chimeras was assembled into the OM. In contrast, no trypsin-resistant fragments reactive with the α-*Ec*BamA antiserum were obtained from cell envelopes containing the Nm_423_Ec or the Nm_480_Ec chimera (Figure 6B, right panel) suggesting that these proteins are not assembled in the OM. These observations were confirmed in urea extraction experiments: both Nm_480_Ec and Nm_423_Ec were fully extractable from the *N. meningitidis* cell envelopes, whereas Ec_423_Nm and Ec_479_Nm did not solubilize in urea (Figure 6C). Thus, only the chimeras containing the C-terminal part of *Nm*BamA appeared to be assembled into the OM of *N. meningitidis*. 

**Figure 7:**
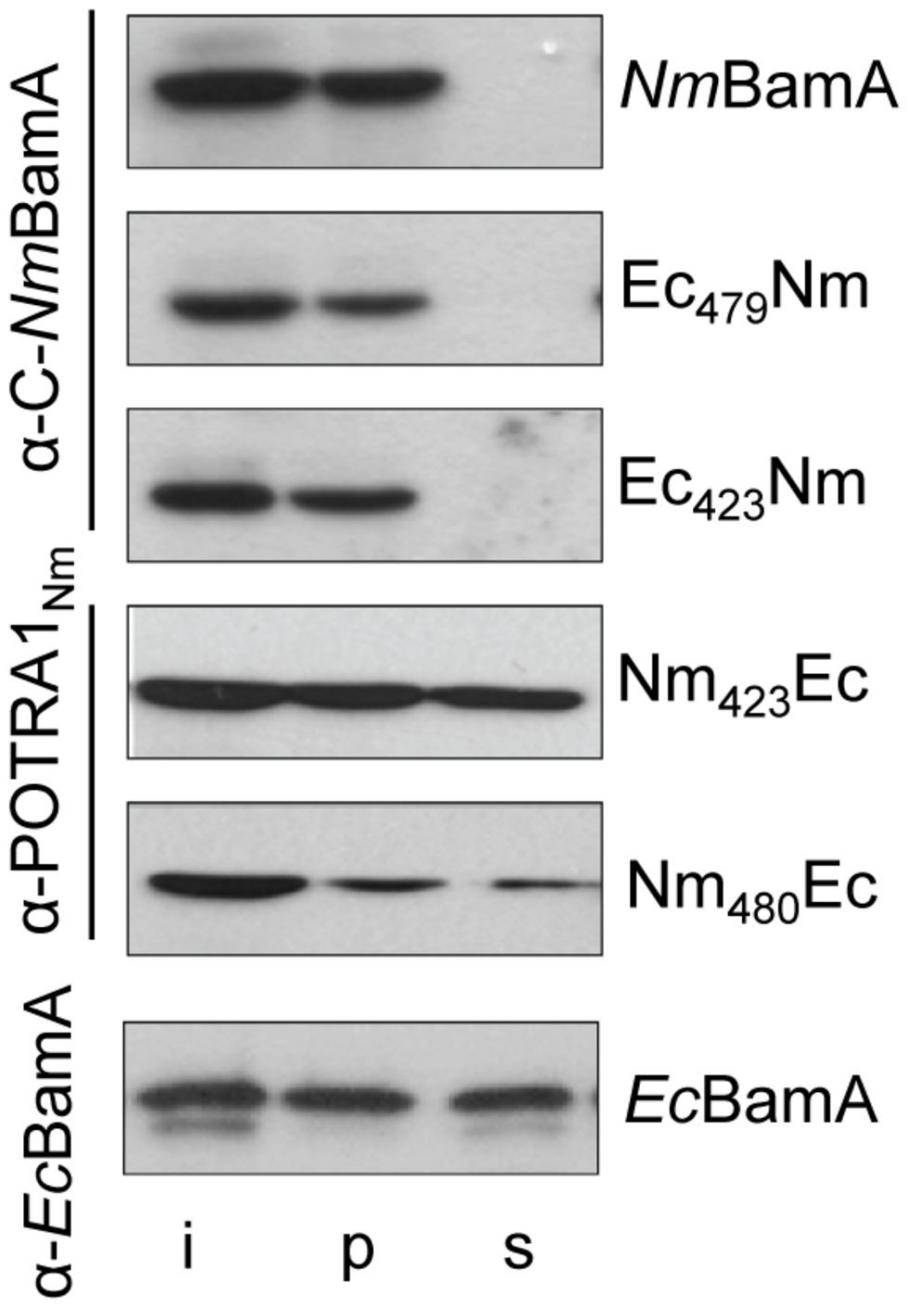
Assembly of BamA chimeras in *E. coli*. Derivatives of *E. coli* strain UTP_BAD_::*bamA* containing plasmids encoding BamA variants were grown in LB containing arabinose and IPTG. Cell envelopes were isolated and extracted with urea. Input (i), urea-insoluble (p) and -soluble (s) fractions were analyzed by SDS-PAGE and immunoblotting with the antibodies indicated on the left. The BamA variants encoded by the plasmids are indicated at the right.

Next, we assessed functionality of the chimeric BamA proteins in *E. coli* by introducing the plasmids containing the chimeric constructs into the *Ec*BamA-depletion strain. All chimeric proteins were produced after growth of the cells in the presence of arabinose plus IPTG (Figure 7). Extraction of the membrane fraction with urea indicated that the chimeric proteins Ec_423_Nm and Ec_479_Nm were fully inserted into the *E. coli* OM, whereas the Nm_480_Ec and Nm_423_Ec proteins were each for ~50% extractable and, therefore, only partially inserted (Figure 7). The plasmid-encoded full-length *Ec*BamA was also partially extracted (Figure 7 bottom panel), in contrast to endogenously expressed chromosomal *Ec*BamA (Figure 1B). Apparently, the cells cannot assemble overexpression levels of *Ec*BamA. Despite significant insertion, none of the chimeras appeared to complement the absence of endogenous *Ec*BamA, since the strains stopped growing in the absence of arabinose and presence of IPTG at 37°C (Figure 2F-I) or at 22°C (Figure S1).

### Analysis of the *Nm*BamA β-barrel Domain

**Figure 8:**
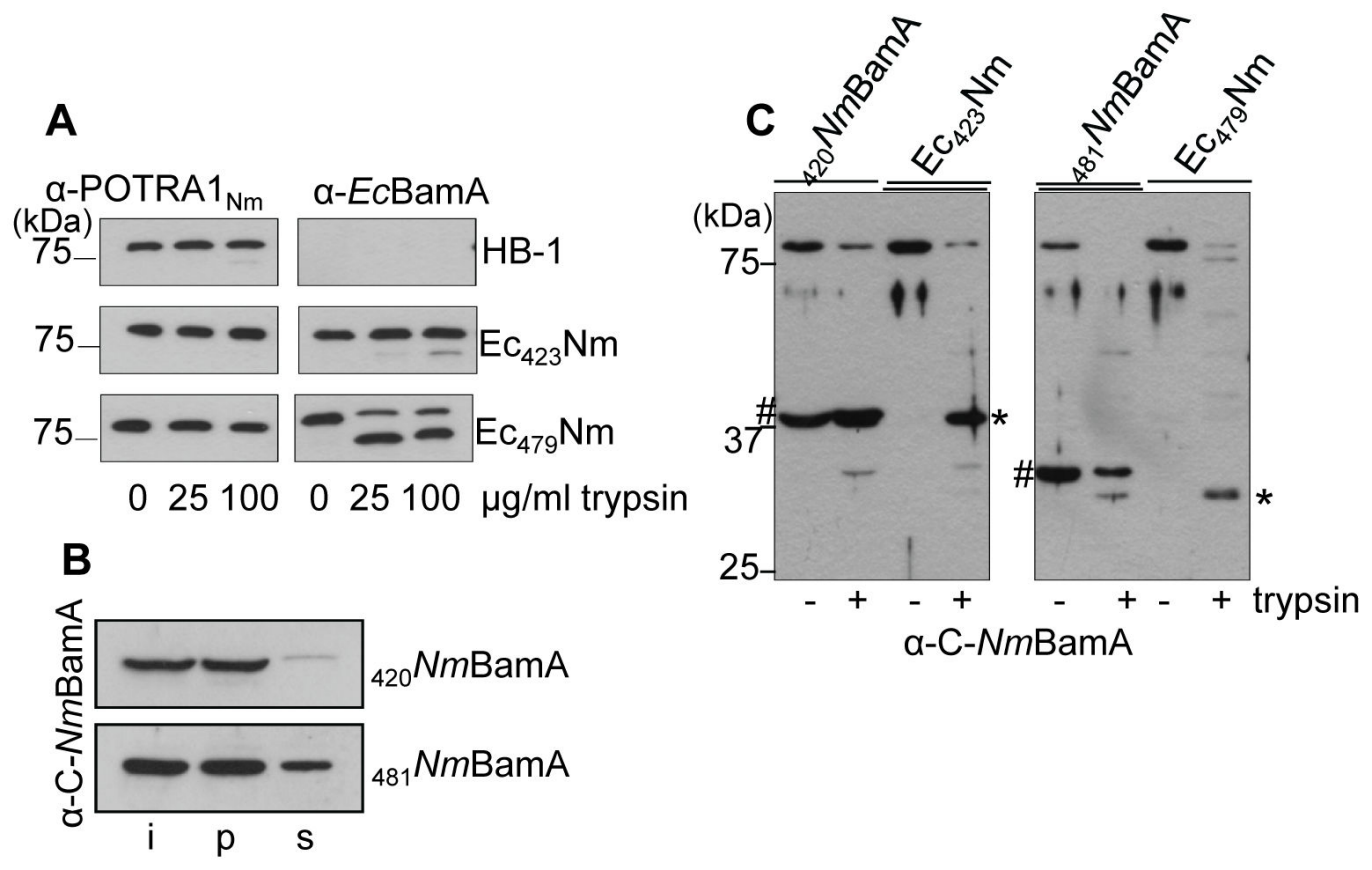
Analysis of the β-barrel domain of *Nm*BamA. A: Intact HB-1 cells and HB-1 cells producing Ec_423_Nm or Ec_479_Nm were treated with the indicated concentrations of trypsin for 15 min and processed for immunoblotting with α-POTRA1_Nm_ (left panels) or α-*Ec*BamA (right panels) antibodies. B. Cell envelopes of HB-1 producing the proteins indicated at the right were treated with urea and processed for immunoblotting with α-C-*Nm*BamA antiserum. C: Cell envelopes of HB-1 producing the proteins indicated on top were treated overnight with or without trypsin and analyzed on immunoblots with α-C-*Nm*BamA antiserum. The # signs indicate the positions of the truncated _420_ *Nm* BamA and _481_ *Nm*BamA variants, wheras the * signs indicate tryptic fragments derived from the chimeric Ec_423_Nm or Ec_479_Nm proteins (similar to those shown in Fig. 6B).

 The simplest model explaining the correct assembly of the C-terminal parts of both the Ec_423_Nm and the Ec_479_Nm chimera into the neisserial OM would be that their membrane-embedded domains are fully comprised within the *Nm*BamA-derived part. These should then have identical conformations. To test this notion, we treated intact cells with limited amounts of trypsin in order to target only the surface-exposed part of the BamA variants. Trypsin treatment of cells of the parent strain HB-1 or HB-1 cells expressing the Ec_423_Nm or Ec_479_Nm chimera did not affect chromosomally encoded wild-type *Nm*BamA as deduced from similar reactivity with Mab α-POTRA1_Nm_, which specifically recognizes POTRA1 of *N. meningitidis* BamA and, therefore, only reacts with endogenous *Nm*BamA (Figure 8A, left panels). Apparently, no readily accessible trypsin-cleavage sites are exposed at the cell surface in native *Nm*BamA. Next, we probed the samples with an antiserum directed against *Ec*BamA to detect specifically the chimeric proteins. Remarkably, an approximately 7-kDa fragment was cleaved off from the majority of the Ec_479_Nm protein whereas little or no cleavage was observed for the Ec_423_Nm chimera (Figure 8A, right panels). Thus, the conformation of the membrane domain of the Ec_479_Nm chimera appears affected in such a way that one of its surface-exposed loops has become accessible to externally added protease. This result indicates that the exchanged fragment between residues 423 and 479 affects the barrel structure and probably belongs to the β-barrel domain, implying that this domain contains 16 β-strands.

 To test whether individual 16- or 12-stranded *Nm*BamA β-barrels could be stably inserted in the OM, we produced such proteins, designated _420_ *Nm*BamA (mature protein starting with amino acid 420 of *Nm*BamA) and _481_ *Nm*BamA (mature protein starting with amino acid 481 of *Nm*BamA), in HB-1. Urea extraction experiments indicated that _420_ *Nm*BamA was completely and _481_ *Nm*BamA was only partially inserted in the OM (Figure 8B). Trypsin treatment of cell envelopes showed that _420_ *Nm*BamA was completely protected from the protease, whereas _481_ *Nm*BamA was for the most part degraded (Figure 8C). The _420_ *Nm*BamA protein migrated at exactly the same position as the trypsin-protected fragment of Ec_423_Nm, suggesting that they possess a similar membrane domain, i.e. a stably integrated 16-stranded β-barrel. 

## Discussion

 In this work we studied the species-specific functioning of BamA, the central component of the OMP insertion machinery, which is essential and conserved in Gram-negative bacteria. Previously, it was reported that the BamA protein of the cyanobacterium *Anabaena* sp. PCC 7120 did not function in *E. coli*, despite its insertion in the *E. coli* OM [31]. This lack of function may be due to the large phylogenetic distance between these bacterial species which is reflected in the distinct pore diameters, differing number of POTRA domains (3 versus 5) and differences in associated complex components for BamA proteins from cyanobacterial and proteobacterial origin [32-35]. We now show that the BamA species specificity extends to much more closely related species, since even BamA homologs of proteobacteria or even within one class of proteobacteria were generally not exchangeable.

Several factors might potentially contribute to this lack of cross-complementation: insufficient assembly of the heterologous BamA into the OM of the host, an inability to associate with the accessory lipoproteins into a fully functional OMP-insertion machinery, or failure of the heterologous BamA to efficiently recognize various OMP substrates. *E. coli* BamA was not assembled into the OM of *N. meningitidis* and therefore could obviously not compensate for the absence of neisserial BamA. In contrast, *N. meningitidis* BamA appeared to be inserted into the *E. coli* OM, at least to a substantial extent. Previously, we showed that neisserial OMPs are only poorly inserted into the *E. coli* OM. This defect was related to the nature of the penultimate amino acid of the OMP: in *Neisseria* this residue is almost invariably a positively charged one, whereas in *E. coli* this is only very rarely the case [23]. Replacing the lysine present in this position in the neisserial porin PorA by glutamine greatly enhanced PorA assembly in *E. coli* [23]. *Nm*BamA is one of the few neisserial OMPs possessing a threonine instead of a positively charged residue at the penultimate position; therefore, it might be sufficiently recognized by the Bam complex to be inserted into the *E. coli* OM. Neisserial BamA was even able to associate in the *E. coli* OM with the essential Bam-complex component BamD but not with BamB. The latter is consistent with the absence of a BamB homolog in *N. meningitidis*[14]. We did not test association of BamC and BamE, but, since these proteins have been shown to interact indirectly with BamA via BamD [13,36], they are likely to form part of the hybrid complex. Thus, despite its proper assembly into the OM and its association with at least the other essential Bam complex component, *Nm*BamA could not functionally replace *Ec*BamA. Therefore, the most likely explanation for its lack of function in *E. coli* is poor recognition of the host’s OMP substrates. 

Paradoxically, whereas the lack of substrate recognition is apparently an issue when BamA proteins are exchanged between different proteobacteria, we recently showed that *E. coli* OMPs could be assembled into the OM of yeast mitochondria [25], and vice versa, that a mitochondrial OMP could be inserted into the *E. coli* OM [26]. However, these assemblies were efficient only when the OMPs were expressed at a low level with extensive amounts of unassembled OMPs accumulating at higher expression levels. It seems likely that also *Nm*BamA can handle *E. coli* OMP substrates to some extent. However, due to the fact that for cellular survival it needs to deal with all OMPs under conditions of suboptimal recognition, it is not unexpected that *Nm*BamA fails to sufficiently handle this load of substrates. Cells then likely die from lethal accumulation of periplasmic OMP aggregates. 

Chimeric proteins comprising BamA domains derived from *N. meningitidis* and *E. coli* were not functional in either host. All evidence indicated that the two chimeras containing the C-terminal part of *N. meningitidis* BamA were inserted into the neisserial OM. However, the conformation of the β-barrel domain of the Ec_479_Nm protein was different as indicated by its accessibility to trypsin in intact cells. The apparent cleavage in one of its extracellular loops also explains the considerably smaller trypsin-resistant fragment detected after trypsinization of the Ec_479_Nm protein in cell envelopes (Figure 6B). In contrast, similar to wild-type *Nm*BamA, the Ec_423_Nm chimera was resistant to trypsin digestion in intact cells. The most straightforward explanation for these results is that the protein segment between residues 423 and 479 does not constitute a hinge region between the POTRA domains and the β-barrel according to the original 12-stranded β-barrel model, but is actually part of the β-barrel. Consequently, in the Ec_479_Nm protein, four β-strands of the *Nm*BamA β-barrel are substituted by the corresponding ones of *Ec*BamA and this substitution results in a conformational change rendering a surface-exposed loop near the C-terminal end sensitive to trypsin. Overall, these data support a 16-stranded rather than the originally proposed 12-stranded membrane β-barrel. Interestingly, during submission of this manuscript the structure of *N. gonorrhoeae* BamA, refolded from solubilized inclusion bodies, was elucidated [37] and found to consist of a large periplasmic domain attached to a 16-stranded β-barrel domain. Thus, our observations from *in vivo* assembled proteins are consistent with the reported BamA structure.

Despite apparently correct membrane insertion of the Ec_423_Nm and Ec_479_Nm chimeras, the proteins could not functionally replace *Nm*BamA. Trypsin digestion of the chimeras in cell envelopes did not yield the intermediate fragments seen for native BamA. These fragments are indicative for association with other Bam complex members [14]. Therefore, the *Ec*BamA-derived POTRA domains in both chimeras apparently fail to associate with the accessory Bam complex components in *N. meningitidis*, which would explain the lack of function of both chimeras in *N. meningitidis*. In addition, it is entirely possible that the *Ec*BamA-moiety of the chimeras does not efficiently recognize the neisserial OMP substrates. In *E. coli*, we expected at least the Ec_423_Nm chimera to function. This hybrid consists of the periplasmic POTRA domains from *Ec*BamA and the entire β-barrel from *Nm*BamA. Like the wild-type *Ec*BamA, it was inserted into the OM of *E. coli* as shown in urea-extraction experiments. With its periplasmic domain derived from *Ec*BamA, this chimeric protein should have no problem recognizing *E. coli* OMP substrates or recruiting accessory Bam components. However, these features were apparently not sufficient to allow for complete functioning. The lack of function of this hybrid suggests that appropriate interactions between the POTRA domains and the β-barrel are essential for function. Indeed, the POTRA5 domain was found to interact with several periplasmic loops in the crystal structure of *Ng*BamA [37]. Such interaction may be essential and not be possible in the case of the Ec_423_Nm hybrid, which could also explain its lack of function.

In conclusion, our results demonstrate high species specificity in the functioning of the BamA component of the OMP assembly machinery. This specificity is most likely related to the requirement for an efficient recognition of the substrate OMPs and subtle species-specific differences in the recognition signals in these substrates. Furthermore, our results obtained with *in vivo* assembled BamA are consistent with the recently reported crystal structure of BamA [37]. Finally, our results indicate that specific interactions between the β-barrel and the POTRA domains might be essential for appropriate functioning.

## Supporting Information

Figure S1 **BamA complementation assays in *E. coli* under slow growth conditions.**
(PDF)

Figure S2 **Multiple sequence alignments of BamA variants.**
(DOCX)

Table S1 **Plasmids used in this study.**
(DOCX)

Table S2 **Primers used in this study.**
(DOCX)
