# Supplementary material for: Species-Specificity of the BamA Component of the Bacterial Outer Membrane Protein-Assembly Machinery
Source: PLoS One. 2013 Dec 20;8(12):e85799. doi: 10.1371/journal.pone.0085799 (PMC3869937; doi:10.1371/journal.pone.0085799)
Supplement: Figure S1 — BamA complementation assays in E. coli under slow growth conditions. (PDF) [file pone.0085799.s001.pdf]

Figure S1

A

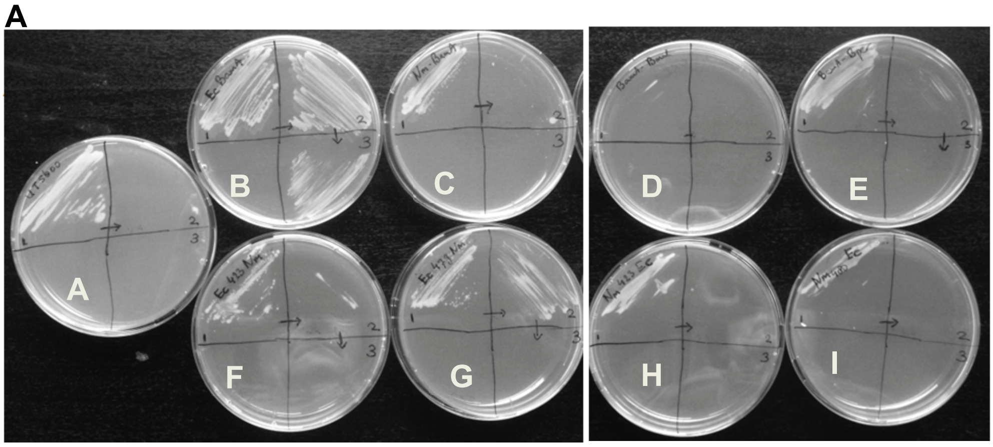

B

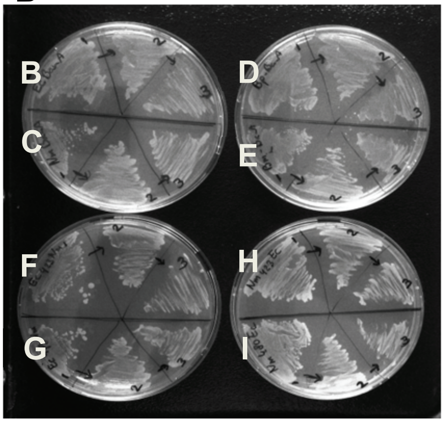

Figure S1: BamA complementation assays in *E. coli* under slow growth conditions.
E. coli strain UTP$_{BAD}$::*bamA* carrying no plasmid (plate A) or plasmids encoding *Ec*BamA (B), *Nm*BamA (C), *Bp*BamA (D), *Bp*BamA (E), Ec$_{423}$Nm (F), Ec$_{479}$Nm (G), Nm$_{423}$Ec (H) or Nm$_{480}$Ec (I) were grown on LB plates containing IPTG without arabinose (panel A) or IPTG plus arabinose (panel B) at 22°C.
Bacteria were restreaked from plates containing arabinose and IPTG onto section 1and subsequently onto section 2. When growth occurred on section 2, cells were further restreaked from section 2 to section 3.
