# Supplementary material for: Species-Specificity of the BamA Component of the Bacterial Outer Membrane Protein-Assembly Machinery
Source: PLoS One. 2013 Dec 20;8(12):e85799. doi: 10.1371/journal.pone.0085799 (PMC3869937; doi:10.1371/journal.pone.0085799)
Supplement: Figure S2 — Multiple sequence alignments of BamA variants. (DOCX) [file pone.0085799.s002.docx]

Figure S2: Multiple sequence alignments of BamA variants

**A**

Ng MKLKQIASALMMLGISPLAFADFTIQDIRVEGLQRTEPSTVFNYLPVKVGDTYNDTHGSA 60

Nm MKLKQIASALMMLGISPLALADFTIQDIRVEGLQRTEPSTVFNYLPVKVGDTYNDTHGSA 60

*******************:****************************************

Ng IIKSLYATGFFDDVRVETADGQLLLTVIERPTIGSLNITGAKMLQNDAIKKNLESFGLAQ 120

Nm IIKSLYATGFFDDVRVETADGQLLLTVIERPTIGSLNITGAKMLQNDAIKKNLESFGLAQ 120

************************************************************

Ng SQYFNQATLNQAVAGLKEEYLGRGKLNIQITPKVTKLARNRVDIDITIDEGKSAKITDIE 180

Nm SQYFNQATLNQAVAGLKEEYLGRGKLNIQITPKVTKLARNRVDIDITIDEGKSAKITDIE 180

************************************************************

Ng FEGNQVYSDRKLMRQMSLTEGGIWTWLTRSDRFDRQKFAQDMEKVTDFYQNNGYFDFRIL 240

Nm FEGNQVYSDRKLMRQMSLTEGGIWTWLTRSNQFNEQKFAQDMEKVTDFYQNNGYFDFRIL 240

******************************::*:.*************************

Ng DTDIQTNEDKTRQTIKITVHEGGRFRWGKVSIEGDTNEVPKAELEKLLTMKPGKWYERQQ 300

Nm DTDIQTNEDKTKQTIKITVHEGGRFRWGKVSIEGDTNEVPKAELEKLLTMKPGKWYERQQ 300

***********:************************************************

Ng MTAVLGEIQNRMGSAGYAYSEISVQPLPNAGTKTVDFVLHIEPGRKIYVNEIHITGNNKT 360

Nm MTAVLGEIQNRMGSAGYAYSEISVQPLPNAETKTVDFVLHIEPGRKIYVNEIHITGNNKT 360

****************************** *****************************

Ng RDEVVRRELRQMESAPYDTSKLQRSKERVELLGYFDNVQFDAVPLAGTPDKVDLNMSLTE 420

Nm RDEVVRRELRQMESAPYDTSKLQRSKERVELLGYFDNVQFDAVPLAGTPDKVDLNMSLTE 420

************************************************************

β1 eL1 β2 β3 eL2 β4

Ng RSTGSLDLSAGWVQDTGLVMSAGVSQDNLFGTGKSAALRASRSKTTLNGSLSFTDPYFTA 480

Nm RSTGSLDLSAGWVQDTGLVMSAGVSQDNLFGTGKSAALRASRSKTTLNGSLSFTDPYFTA 480

************************************************************

β5 eL3 β6 β7

Ng DGVSLGYDIYGKAFDPRKASTSVKQYKTTTAGGGVRMGIPVTEYDRVNFGLAAEHLTVNT 540

Nm DGVSLGYDVYGKAFDPRKASTSIKQYKTTTAGAGIRMSVPVTEYDRVNFGLVAEHLTVNT 540

********:*************:*********.*:**.:************.********

eL4 β8 β9

Ng YNKAPKRYADFIRKYGKTDGADGSFKGLLYKGTVGWGRNKTDSASWPTRGYLTGVNAEIA 600

Nm YNKAPKHYADFIKKYGKTDGTDGSFKGWLYKGTVGWGRNKTDSALWPTRGYLTGVNAEIA 600

******:*****:*******:****** **************** ***************

eL5 β10 β11 eL6

Ng LPGSKLQYYSATHNQTWFFPLSKTFTLMLGGEVGIAGGYGRTKEIPFFENFYGGGLGSVR 660

Nm LPGSKLQYYSATHNQTWFFPLSKTFTLMLGGEVGIAGGYGRTKEIPFFENFYGGGLGSVR 660

************************************************************

β12 β13

Ng GYESGTLGPKVYDEYGEKISYGGNKKANVSAELLFPMPGAKDARTVRLSLFADAGSVWDG 720

Nm GYESGTLGPKVYDEYGEKISYGGNKKANVSAELLFPMPGAKDARTVRLSLFADAGSVWDG 720

************************************************************

eL7 β14 β15

Ng RTY----TAAENGNNKSVY-SENAHKSTFTNELRYSAGGAVTWLSPLGPMKFSYAYPLKK 775

Nm KTYDDNSSSATGGRVQNIYGAGNTHKSTFTNELRYSAGGAVTWLSPLGPMKFSYAYPLKK 780

:** ::* .*. :.:* : *:************************************

eL8 β16

Ng KPEDEIQRFQFQLGTTF 792

Nm KPEDEIQRFQFQLGTTF 797

*****************

**B**

Ng ------------MKLKQIASALMMLG-ISPLAFADFTIQDIRVEGLQRTEPSTVFNYLPV 47

Nm ------------MKLKQIASALMMLG-ISPLALADFTIQDIRVEGLQRTEPSTVFNYLPV 47

Bm -----MLFKPHRFVPKTVAAAALAAHGLAAHATAPFVVQDIKIEGLQRVEAGSVFAYLPI 55

Bp MSLRRMFHHKKGVIPGLLAAALLAPA--LAHAFEPFVVRDIRVEGIQRTDAGTVFGYLPV 58

Ec ------------MAMKKLLIASLLFSSATVYGAEGFVVKDIHFEGLQRVAVGAALLSMPV 48

. : * : . *.::**:.**:**. .:.: :*:

Ng KVGDTYNDTHGSAIIKSLYATGFFDDVRVETADGQLLLTVIERPTIGSLNITGAKMLQND 107

Nm KVGDTYNDTHGSAIIKSLYATGFFDDVRVETADGQLLLTVIERPTIGSLNITGAKMLQND 107

Bm KQGDTFTDGKASEAIRALYATGFFNDVRIATQGGVVIVQVQERPAIASIDFTGIKEFDKD 115

Bp KVGEKFTDEEATEAVRRLYGTGFFSDVQIQTDNNVVVVVVQERPTIASISFNGMREFDSK 118

Ec RTGDTVNDEDISNTIRALFATGNFEDVRVLRDGDTLLVQVKERPTIASITFSGNKSVKDD 108

: *:. .* . : :: *:.** *.**:: .. ::: * ***:*.*: :.* : ....

Ng AIKKNLESFGLAQSQYFNQATLNQAVAGLKEEYLGRGKLNIQITPKVTKLARNRVDIDIT 167

Nm AIKKNLESFGLAQSQYFNQATLNQAVAGLKEEYLGRGKLNIQITPKVTKLARNRVDIDIT 167

Bm NLNKALKAVGLSQGRYYDKALVDKAEQELKRQYLTRGFYAAEVSTTVTPVDANRVSILFA 175

Bp AITKSLAQVGFGEGRIFDQSMLERAEYELKEQYLAKGKYGVEVTATVTPLPRNRVGVSFD 178

Ec MLKQNLEASGVRVGESLDRTTIADIEKGLEDFYYSVGKYSASVKAVVTPLPRNRVDLKLV 168

:.: * *. .. ::: : *: * * .:.. ** : ***.: :

Ng IDEGKSAKITDIEFEGNQVYSDRKLMRQMSLTEGGIWTWLTRSDRFDRQKFAQDMEKVTD 227

Nm IDEGKSAKITDIEFEGNQVYSDRKLMRQMSLTEGGIWTWLTRSNQFNEQKFAQDMEKVTD 227

Bm VAEGPSAKIRQINFIGNKAFKTSTLRDEMQLSTPNWFSWYTKNDLYSKEKLTGDLENVRS 235

Bp VFEGEVAKIREIRVVGSKAFSEGELLDQFDLTTPGWLTWYTNTDKYSREKLEGDIERLRS 238

Ec FQEGVSAEIQQINIVGNHAFTTDELISHFQLRDEVPWWNVVGDRKYQKQKLAGDLETLRS 228

. ** *:* :*.. *.:.:. * .:.* . :..:*: *:* : .

Ng FYQNNGYFDFRILDTDIQTNEDKTRQTIKITVHEGGRFRWGKVSIEGDTNEVPKAELEKL 287

Nm FYQNNGYFDFRILDTDIQTNEDKTKQTIKITVHEGGRFRWGKVSIEGDTNEVPKAELEKL 287

Bm YYLNRGYLEFNIESTQVSISPDKKDMYLTVALHEGEPYTVSSVKLAGNLLDR-QAELEKL 294

Bp FYLDQGYLEFTVEPPQVTISPDRKDIYITITVHEGEPYKVREVKLAGNLMGL-DSEINNL 297

Ec YYLDRGYARFNIDSTQVSLTPDKKGIYVTVNITEGDQYKLSGVEVSGNLAGH-SAEIEQL 287

:* :.** * : .:: . *:. :.: : ** : *.: *: .:*:::*

Ng LTMKPGKWYERQQMTAVLGEIQNRMGSAGYAYSEISVQPLPNAGTKTVDFVLHIEPGRKI 347

Nm LTMKPGKWYERQQMTAVLGEIQNRMGSAGYAYSEISVQPLPNAETKTVDFVLHIEPGRKI 347

Bm VKIKPGDRFSAEKLQQTTKAIVDKLGQYGYAFATVNAQPEIDQATHKVGLTLVVDPSRRV 354

Bp VEIKPGEVFSAAKANNSAKAITNYLGDLGYAFANVNPNPQLDRAKHEADVTFYVDPSRRV 357

Ec TKIEPGELYNGTKVTKMEDDIKKLLGRYGYAYPRVQSMPEINDADKTVKLRVNVDAGNRF 347

::**. :. : * . :* ***:. :. * : : . . . ::...:.

Ng YVNEIHITGNNKTRDEVVRRELRQMESAPYDTSKLQRSKERVELLGYFDNVQFDAVPLAG 407

Nm YVNEIHITGNNKTRDEVVRRELRQMESAPYDTSKLQRSKERVELLGYFDNVQFDAVPLAG 407

Bm YVRRINIVGNTRTRDEVVRREMRQLESSWFDSSRLALSKDRVNRLGYFTDVDVTTVPVEG 414

Bp YVRRIQIGGNTRTRDEVVRREMRQQEAAWYDAGDIKVSRDRVDRLGYFNEVNVKTDPVPG 417

Ec YVRKIRFEGNDTSKDAVLRREMRQMEGAWLGSDLVDQGKERLNRLGFFETVDTDTQRVPG 407

**..*.: ** ::* *:***:** *.: .:. : .::*:: **:* *: : : *

β1 eL1 β2 β3 eL2

Ng TPDKVDLNMSLTERSTGSLDLSAGWVQDTGLVMSAGVSQDNLFGTGKSAALRASRSKTTL 467

Nm TPDKVDLNMSLTERSTGSLDLSAGWVQDTGLVMSAGVSQDNLFGTGKSAALRASRSKTTL 467

Bm TNDQVDVNVKVAEKPTGAITLGAGFSSTDKVVLSAGISQDNVFGSGTSLAVNVNTAKSYR 474

Bp SPDQVDVNVDVKEKPTGIINLGVGYGSSEKAILSAGISEDNVFGSGTNLTLQLNTSKTNR 477

Ec SPDQVDVVYKVKERNTGSFNFGIGYGTESGVSFQAGVQQDNWLGTGYAVGINGTKNDYQT 467

: *:**: .: *: ** : :. *: :.**:.:** :*:* :. . .

β4 β5 eL3 β6

Ng NGSLSFTDPYFTADGVSLGYDIYGKAFDPRKASTSVKQYKTTTAGGGVRMGIPVTEYDRV 527

Nm NGSLSFTDPYFTADGVSLGYDVYGKAFDPRKASTSIKQYKTTTAGAGIRMSVPVTEYDRV 527

Bm TLTVTQVDPYFTVDGIKRITDVFYRTYQPLYYSTNS-SFRIITAGGNLKFGIPFSETDTV 533

Bp AVVLSHTDPYFTKDGISRTTSAYYRVTEP-WDNNDG-DYRVKAMGLGMNFGVPISEYDRI 535

Ec YAELSVTNPYFTVDGVSLGGRLFYNDFQA--DDADLSDYTNKSYGTDVTLGFPINEYNSL 525

:: .:**** **:. : . :. . . .: : * .: :..*..* : :

β7 eL4 β8

Ng NFGLAAEHLTVN--TYNKAPKRYADFIRKYGKTDGADGSFKGLLYKGTVGWGRNKTDSAS 585

Nm NFGLVAEHLTVN--TYNKAPKHYADFIKKYGKTDGTDGSFKGWLYKGTVGWGRNKTDSAL 585

Bm YFGAGFEQNRLD--VDSNTPQSYQDYVNEFGRVS------NTVPLT--IGWSRDARDSAL 583

Bp FLGGTFERNQID--LYNNSPQAYRDFVDQYGNST------NALIFN--TGWSKDTRDSAL 585

Ec RAGLGYVHNSLSNMQPQVAMWRYLYSMGEHPSTSDQDNSFKTDDFTFNYGWTYNKLDRGY 585

* : :. . : * : :. : . ** : * .

β9 eL5 β10 β11

Ng WPTRGYLTGVNAEIALPGSKLQYYSATHNQTWFFPLSKTFTLMLGGEVGIAGGYGRT-KE 644

Nm WPTRGYLTGVNAEIALPGSKLQYYSATHNQTWFFPLSKTFTLMLGGEVGIAGGYGRT-KE 644

Bm IPSRGYFTQANAEYGVPVGKIQYYKMDVQGQYYYSFARGFILGLNFQAGYGNGIG---NP 640

Bp APTKGAYTRLKGDFST--MDLKYYLLTAQQQYYLPLGRSYTLALNGMIDYGRSYGG--LD 641

Ec FPTDGSRVNLTGKVTIPGSDNEYYKVTLDTATYVPIDDDHKWVVLGRTRWGYGDGLGGKE 645

*: * . ... . :** : : .: . : . . *

eL6

Ng IPFFENFYGGGLGSVRGYESGTLGPKVY------------------------DEYGEKIS 680

Nm IPFFENFYGGGLGSVRGYESGTLGPKVY------------------------DEYGEKIS 680

Bm YPIFKNYYAGGIGSVRGYEPSSLGPR---------------------------DTKTNDP 673

Bp YPVIKNVYAGGIGTVRGYEGASLGPR---------------------------DRLTGDY 674

Ec MPFYENFYAGGSSTVRGFQSNTIGPKAVYFPHQASNYDPDYDYECATQDGAKDLCKSDDA 705

*. :* *.** .:***:: ::**:

β12 β13

Ng YGGNKKANVSAELLFPMP--GAKDARTVRLSLFADAGSVWDGRTY----TAAENGNNKSV 734

Nm YGGNKKANVSAELLFPMP--GAKDARTVRLSLFADAGSVWDGKTYDDNSSSATGGRVQNI 738

Bm IGGSKMVVGNIELTFPLP--GTGYDRTLRVFTFLDGGNVWGNAPG-----GTSTG----- 721

Bp IGGSRRMVANAQLYLPFP--GASKDRTLRWFVFTDAGQVAAGSGM-----SCTAGKPDSE 727

Ec VGGNAMAVASLEFITPTPFISDKYANSVRTSFFWDMGTVWDTNWD----SSQYSGYPDYS 761

**. . :: * * . .::* * * * * . *

eL7 β14 β15 eL8 β16

Ng Y-SENAHKSTFTNELRYSAGGAVTWLSPLGPMKFSYAYPLKKKPEDEIQRFQFQLGTTF 792

Nm YGAGNTHKSTFTNELRYSAGGAVTWLSPLGPMKFSYAYPLKKKPEDEIQRFQFQLGTTF 797

Bm -----------ANGLRYGYGIGLAWISPIGPLKLSLGFPLQKHEGDQYQKFQFQIGTAF 769

Bp V--------EDPCGWRFSAGIGLSWQSPLGPLQLSYARPLNSKSGDDTQAFQFQIGTGF 778

Ec D----------PSNIRMSAGIALQWMSPLGPLVFSYAQPFKKYDGDKAEQFQFNIGKTW 810

. * . * .: * **:**: :* . *::. *. : ***::*. :

A, B: Sequence and secondary structure alignments for BamA homologs based on the crystal structure of *Ng*BamA (1).

The individual POTRA domains (POTRA 1-5) are indicated in cyan, red, green, purple, and blue, respectively. Beta-strands in the C-terminal β-barrel domain are indicated in gray. Secondary structural elements are numbered within the respective domain. β: β-strand; eL: extracellular loop. Ng, *Neisseria gonorrhoeae*; Nm, *N. meningitidis*; Bm, *Burkholderia mallei*; Bp, *Bordetella pertussis*, Ec, *Escherichia coli*

1. Noinaj N, Kuszak AJ, Gumbart JC, Lukacik P, Chang H, Easley NC, Lithgow T, Buchanan SK (2013) Structural insight into the biogenesis of β-barrel membrane proteins. Nature 501: 385-390.
