# Supplementary material for: Species-Specificity of the BamA Component of the Bacterial Outer Membrane Protein-Assembly Machinery
Source: PLoS One. 2013 Dec 20;8(12):e85799. doi: 10.1371/journal.pone.0085799 (PMC3869937; doi:10.1371/journal.pone.0085799)
Supplement: Table S1 — Plasmids used in this study. (DOCX) [file pone.0085799.s003.docx]

Table S1 Plasmids used in this study

| Plasmid | Characteristics^a^ | Source |
| --- | --- | --- |
| pRV1300 | Nm*bamA::kan* gene replacement construct | [1] |
| pCRII-TOPO | TA-cloning vector | Invitrogen |
| pFP10-c-*lbpA* | *Neisseria* plasmid containing the *lbpA* gene behind an IPTG-inducible promoter | [2] |
| pFP10-Nm*bamA* | pFP10 carrying Nm*bamA* fused to the fragment of *lbpA* encoding the signal sequence | This study |
| pFP10-Ng*bamA* | pFP10 carrying Ng*bamA* fused to the fragment of *lbpA* encoding the signal sequence | This study |
| pFP10-Bm*bamA* | pFP10 carrying Bm*bamA* fused to the fragment of *lbpA* encoding the signal sequence | This study |
| pFP10-Bp*bamA* | pFP10 carrying Bp*bamA* fused to the fragment of *lbpA* encoding the signal sequence | This study |
| pFP10-Ec*bamA* | pFP10 carrying Ec*bamA* fused to the fragment of *lbpA* encoding the signal sequence | This study |
| pFP10-*Ec_479_Nm* | pFP10 carrying a chimera encoding the LbpA signal sequence and amino acids 21-479 of *Ec*BamA fused to amino acids 480-797 of *Nm*BamA | This study |
| pFP10-*Nm_480_Ec* | pFP10 carrying a chimera encoding the LbpA signal sequence and amino acids 22-480 of *Nm*BamA fused to amino acids 481-810 of *E*cBamA | This study |
| pFP10-*Ec_423_Nm* | pFP10 carrying a chimera encoding the LbpA signal sequence and amino acids 21-423 of *Ec*BamA fused to amino acids 424-797 of *Nm*BamA | This study |
| pFP10-*Nm_423_Ec* | pFP10 carrying a chimera encoding the LbpA signal; sequence and amino acids 22-423 of *Nm*BamA fused to amino acids 424-810 of *Ec*BamA | This study |
| pFP10-_420_Nm*bamA* | pFP10 encoding the C-terminal 378 amino acids of *Nm*BamA fused to the LbpA signal sequence | [3] |
| pFP10-_481_Nm*bamA* | pFP10 encoding the C-terminal 378 amino acids of *Nm*BamA fused to the LbpA signal sequence | This study |
| pEN11-His-Omp85 | pFP10-derived plasmid encoding N-terminally His-tagged *Nm*BamA | [4] |
| pRV2000 | pBR322-based plasmid, encoding *Nm*BamA under the control of an IPTG-inducible promoter | [1] |
| pRV-His-*Nm*BamA | pRV2000 encoding N-terminally His-tagged *Nm*BamA | This study |

^a^ *Ec, E. coli*; *Nm*, *N. meningitidis*; *Ng, N. gonorrhoeae*; *Bm, B. mallei*; *Bp, B. pertussis*

1. Voulhoux R, Bos MP, Geurtsen J, Mols M, Tommassen J (2003) Role of a highly conserved bacterial protein in outer membrane protein assembly. Science 299: 262-265.
2. Pettersson A, Kortekaas J, Weynants VE, Voet P, Poolman JT, et al. (2006) Vaccine potential of the *Neisseria meningitidis* lactoferrin-binding proteins LbpA and LbpB. Vaccine 24: 3545-3557.
3. Bos MP, Robert V, Tommassen J (2007) Functioning of outer membrane protein assembly factor Omp85 requires a single POTRA domain. EMBO Rep 8:1149-1154.
4. Volokhina EB, Beckers F, Tommassen J, Bos MP (2009). The β-barrel assembly complex of *Neisseria meningitidis.* J Bacteriol 191: 7074-7085.
