# Supplementary material for: Species-Specificity of the BamA Component of the Bacterial Outer Membrane Protein-Assembly Machinery
Source: PLoS One. 2013 Dec 20;8(12):e85799. doi: 10.1371/journal.pone.0085799 (PMC3869937; doi:10.1371/journal.pone.0085799)
Supplement: Table S2 — Primers used in this study. (DOCX) [file pone.0085799.s004.docx]

Table S2. Primers used in this study. Restriction sites used for cloning are underlined.

| Primer name | Sequence | Restriction site |
| --- | --- | --- |
| BmF | ATCATATGCAACGGCGCCCTTCG | NdeI |
| BmR | ATGACGTCTCAGAACGCCGTCC |  |
| BpF | ATCATATGCCTTCGAGCCCTTTGT | *Nde*I |
| BpR | ATGACGTCTCAGAAACCCGTGCC |  |
| NmF | ATCATATGCCGACTTCACCATCCA | *Nde*I |
| NmR | ATGACGTCTTAGAACGTCGTGCC | *Aat*II |
| EcF | ATCATATGCCGCTGAAGGGTTCGTAGTGAAAGAT | *Nde*I |
| EcR | ATGACGTCTTACCAGGTTTTACCGATGTTA | *Aat*II |
| Omp85-F481 | ATCATATGCCGACGGGGTCAGCCTGGGCTAC | *Nde*I |
| HingeF | CCGTACTTCACGGCAGACGGCGTCAGCCTGGG |  |
| EcNmF | GTAAAAGAGCGCAACACCGGTTCCCTGGATTTGAGCGCG |  |
| NmEcR | CCAAAGTTGAAGCTACCGGTGGAACGTTCGGTCAGACTC |  |
| HingeR | CCCAGGCTGACGCCGTCTGCCGTGAAGTACGG |  |
| Omp85R2 | CAAAGAAGGGGATTTCTTTG |  |
| Omp85F3 | GGCGGACGTTTCCGTTGGGG |  |
| Omp85F4 | atacctcgggcgcggcaaac |  |
| Omp85R7 | gcccggttcgatgtgcaggacgaa |  |
| G | TTTGCCGTCTGAACCCTTTAAAATCACAACCGTTGCCGG |  |
| Q-for-1 | TTTTCCGCTTACCCTGACTG |  |
| Q-for-2 | GTAATACCATTCGCGCTCTG |  |
| Q-for-3 | TCCGTCATATGCCTTCGAG |  |
| Q-rev-Ngo | GTACGCTGCAAGCCTTCG |  |
| Q-rev-Eco | TGGTCGGACGTTCTTTTACC |  |
| Q-rev-Bmal | GCAAGCCTTCGATCTTGATG |  |
| Q-rev-Bper | ACCTTGACCGGCAGGTAG |  |
